# Fluorophore multimerization on a PEG backbone as a concept for signal amplification and lifetime modulation

**DOI:** 10.1038/s41598-024-62548-4

**Published:** 2024-05-24

**Authors:** Thorge Reiber, Oskar Hübner, Christian Dose, Dmytro A. Yushchenko, Ute Resch-Genger

**Affiliations:** 1 Department of Chemical Biology, Miltenyi Biotec B.V. & Co. KG, Friedrich-Ebert-Straße 68, 51429 Bergisch Gladbach, Germany; 2 Division Biophotonics, Federal Institute for Materials Research and Testing (BAM), Richard‑Willstaetter‑Str. 11, 12489 Berlin, Germany; 3 Department of Chemistry, Humboldt-Universität zu Berlin, Brook-Taylor-Str. 2, 12489 Berlin, Germany

**Keywords:** Label, Multimeric dyes, Flow cytometry, Fluorescence microscopy, Fluorescence lifetime imaging (flim), Brightness, Photostability, Photochemistry, Physical chemistry

## Abstract

Fluorescent labels have strongly contributed to many advancements in bioanalysis, molecular biology, molecular imaging, and medical diagnostics. Despite a large toolbox of molecular and nanoscale fluorophores to choose from, there is still a need for brighter labels, e.g., for flow cytometry and fluorescence microscopy, that are preferably of molecular nature. This requires versatile concepts for fluorophore multimerization, which involves the shielding of dyes from other chromophores and possible quenchers in their neighborhood. In addition, to increase the number of readout parameters for fluorescence microscopy and eventually also flow cytometry, control and tuning of the labels’ fluorescence lifetimes is desired. Searching for bright multi-chromophoric or multimeric labels, we developed PEGylated dyes bearing functional groups for their bioconjugation and explored their spectroscopic properties and photostability in comparison to those of the respective monomeric dyes for two exemplarily chosen fluorophores excitable at 488 nm. Subsequently, these dyes were conjugated with anti-CD4 and anti-CD8 immunoglobulins to obtain fluorescent conjugates suitable for the labeling of cells and beads. Finally, the suitability of these novel labels for fluorescence lifetime imaging and target discrimination based upon lifetime measurements was assessed. Based upon the results of our spectroscopic studies including measurements of fluorescence quantum yields (QY) and fluorescence decay kinetics we could demonstrate the absence of significant dye-dye interactions and self-quenching in these multimeric labels. Moreover, in a first fluorescence lifetime imaging (FLIM) study, we could show the future potential of this multimerization concept for lifetime discrimination and multiplexing.

## Introduction

In the last years, a large toolbox of fluorescent labels and reporters has been developed for the labeling of biomolecules and particles, for applications in immunoassays, cell biology, medical diagnostics, and bioimaging^1^. This includes small organic dyes and different types of larger nanoparticles (NPs) such as semiconductor and lanthanide nanocrystals, carbon-based nanomaterials, NPs made from semiconducting polymers, fluorophore-doped or labeled inorganic or organic polymeric NPs as well as dyes displaying aggregation-induced emission (AIE)^1–10^. The suitability of fluorescent labels, e.g., for biomarker, cell, and tissue analysis depends on their optical properties and their photostability as well as their hydrophilicity. Optical properties relevant for reporter choice include the spectral position and width of the absorption and emission bands and the Stokes shift, and brightness (B). The latter presents the product of the label´s molar absorption coefficient or absorption cross section at the chosen excitation wavelength and its photoluminescence quantum yield (QY) and determines the size of the measured luminescence signals from the sample side^4^. For fluorescence microscopy and imaging applications, also photostability is important as photostable labels can be measured at prolongated illumination times to increase the number of emitted photons and hence the fluorescence signals^1^. For target-specific labeling and the preparation of bioconjugates such as fluorophore-labeled antibodies, functional groups compatible with common bioconjugation strategies are essential. Ideally, conjugation is done via the site-specific attachment of the fluorescent label to the target-specific recognition moiety or binder without hampering its function^11–14^. In principle, nanoparticles (NPs) can provide larger luminescence signals because of their commonly higher brightness compared to molecular fluorophores and meanwhile, different strategies to interface NPs with biomolecules have been reported^15^. However, NPs represent complex systems with a size, ligand, and surface group distribution that are less well-defined than molecular structures^4^. This can result in heterogeneous bioconjugates and can affect conjugate binding specificity^16,17^. Moreover, the analytical characterization of NP-bioconjugates is more tedious and requires more advanced methods compared to molecular systems^18^. In addition, their colloidal nature makes purification more challenging. Therefore, for most applications of fluorescence methods like flow cytometry and fluorescence microscopy, presently in the health sector, mainly molecular labels are used.

The strong interest in pushing detection sensitivity to its ultimate limits, e.g., for studies of low-expressed targets and low-affinity binders, calls for novel fluorescent labels with an improved brightness and a high photostability compared to currently utilized organic dyes. B values of most small molecular dyes employed for flow cytometry and fluorescence microscopy range from 7 × 10^4^ to 2 × 10^5^ M^−1^ cm^−1^. Larger B values can be realized with phycobiliprotein proteins which have molar absorption coefficients > 10^6^ M^−1^ cm^−1^ and QY > 80%^19^. This is achieved by combining several bilin chromophores in a single emitter. The search for brighter labels for flow cytometry and fluorescence microscopy initiated other approaches of fluorophore multimerization. Challenging is here the control of dye-dye interactions that can result in the formation of non- or barely emissive H-type dimers or aggregates followed by fluorescence quenching via homo-FRET^20–22^. Dye aggregation is usually undesired except some specific applications like, e.g. for the design of enzyme substrates^23^ or activatable fluorescent probes^24^. Many common dye classes such as cyanines, xanthenes, and BODIPYs are prone to aggregation-induced fluorescence which can considerably limit the brightness of the resulting multimeric labels and bioconjugates for common multimerization concepts. Hence, versatile strategies are needed that enable the controlled multimerization of a large number of non-interacting, “shielded”, and unquenched dyes for different dye classes and provide control of the spatial arrangement and orientation of the dyes in such multi-chromophoric structures^19^. Up to now, this has been mainly assessed for DNA scaffolds including DNA origami structures and dendrimeric DNA nanostructures that can be made in different sizes and shapes^25–29^. However, for such DNA-based systems, structure-dependent interactions of dye and DNA have been observed which can affect the dynamics of the formed nanostructures^30^ as well as rather low stabilities to nucleases for use in live-cell imaging^31^.

**Figure 1:**
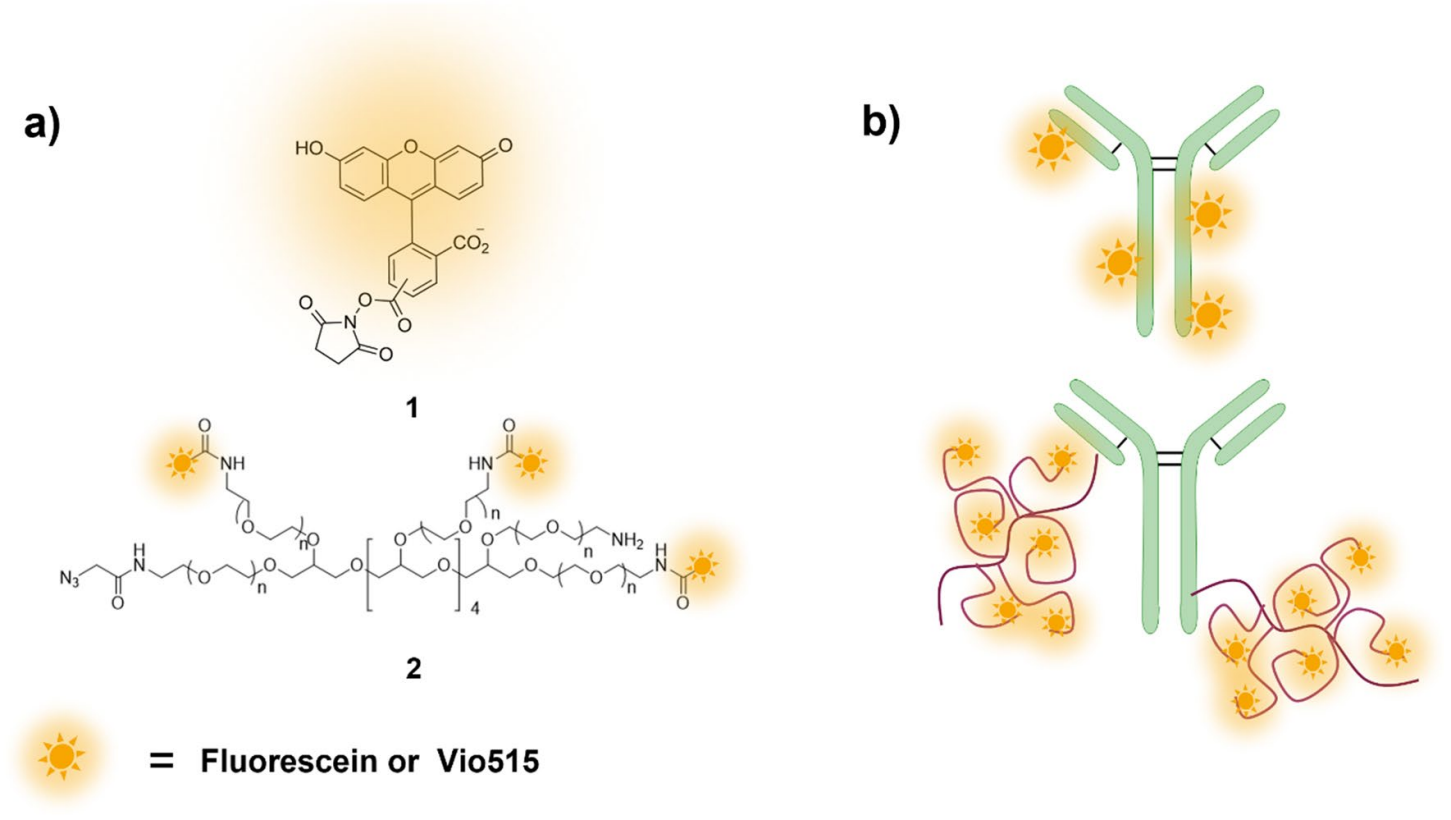
Chemical structures and schematic representation of (**a**) Fam (5/6-Carboxyfluorescein) succinimidyl ester **1** and PEG intermediates **2** used in this study; (**b**) antibody conjugates prepared either via conjugation with a monomeric fluorophore or with its multimeric PEG intermediates. The fluorophores are depicted as glowing stars.

In the present work, we assessed a simple and effective approach for fluorophore multimerization based on a polyethylene glycol (PEG) scaffold for the suppression of dye-dye interactions and fluorophore self-quenching similar to VioBright (VB) dyes developed at Miltenyi^32^. The small dye-polymer structures are schematically presented in Fig. 1. Thereby, we systematically explored the spectroscopic properties of the multimeric dyes VioBright-Fam and VioBright-Vio515 and their monomeric counterparts Fam and Vio515. Antibody conjugates prepared based on multimeric and monomeric dyes were used for cells and beads labeling and studied by means of steady state and time resolved fluorometry as well as flow cytometry and fluorescence microscopy. Subsequently, the applicability of these dyes for lifetime discrimination and multiplexing in conjunction with fluorescence lifetime imaging (FLIM) was examined in a first proof-of-concept experiment by distinguishing epitopes stained with labels based on multimeric and monomeric fluorophores for CD4 +/CD8 + cells as well as beads selectively binding to the Fc regions of antibodies. Overall, our results confirm the superior performance and huge application potential of these novel multimeric reporters and their ease of use.

## Materials and methods

### Materials

All recombinant antibodies and Vio515 NHS ester were produced by *Miltenyi Biotec* (Bergisch Gladbach, Germany). 5/6-Carboxyfluorescein NHS ester was purchased from *Thermo Fisher* (Waltham, MA, USA). 20 kDa 8-arm PEG Amine (hexaglycerol core) HCl salt was purchased from *JenKem* (Beijing, China). Azidoacetic acid NHS ester and DBCO-PEG_4_ NHS ester were purchased from *Jena Bioscience* (Jena, Germany). All reagents were used without further purification or analysis. Phosphate buffered saline (PBS) pH 7.4 was produced by *Miltenyi Biotec*. Carbonate buffer pH 8.3 and 500 mM carbonate buffer pH 9.0 were prepared once by using desalted and sterile-filtered water. PEB buffer was prepared with PBS pH 7.4, 5 mM EDTA and 0.5% bovine serum albumin. Dimethyl sulfoxide was purchased from *Thermo Fisher*. SUP-T1 cells were cultivated in Roswell Park Memorial Institute (RPMI 1640) medium from *Biowest* (Nuaillé, France) supplemented with 10% fetal calf serum (FCS) from *Biochrom* (Berlin, Germany) and 2 mM L-glutamine from *Lonza* (Basel, Switzerland). The cells were maintained at 37 °C and 5% CO_2_.

### Bioconjugation

The preparation of the antibody-dye conjugates and the intermediates is described in detail in the Supporting Information (SI).

### Product purification

Obtained intermediates as well as antibody-dye conjugates were purified using NAP columns from *Cytiva Lifescience* (Marlborough, MA, USA) pre-packed with Sephadex G-25 Grade DNA resin. Final conjugates were purified on an ÄKTA pure 25 chromatography system equipped with a Superdex 200 Increase 10/300 GL column from *Cytiva Lifesciences*. Elution was done with PBS pH 7.4 at a flow rate of 0.75 mL/min and fraction size of 0.5 mL. Detection was done photometrically, measuring the absorbances at 280 nm (antibodies) and 495 nm (Fam, Vio515).

### Surface staining of cells and beads

The concentration of the antibody conjugates was adjusted to 50 µg/ml in PBS and the antibody conjugates were incubated with fixed cells or beads for 10 min at RT in the dark resulting in final concentrations of unbound conjugates between 0.1 and 10 µg/mL (also referred to as low and high labeling densities) binding with the cells/beads bearing the respective targets on their surface. The labeled cells/beads were centrifuged (2000 rpm, 5 min), the staining solution was removed, and the labeled cells/beads were resuspended in PBS buffer. The staining was performed in Eppendorf tubes and samples pipetted into either 96-well plate for flow cytometry analysis or 24-well glass plate for confocal microscopy.

### Flow cytometry

Flow cytometry data were obtained with a MACSQuant X Analyzer from *Miltenyi Biotec* with 488 nm laser excitation, emission detection in B1 channel and medium flow rate. 400,000 cells/well in a 96 well-plate were stained and 50.000 events recorded at medium flow rate. Data analysis was carried out using MACSQuantify software version 2.13.0. The gating strategy is described in detail in the SI and highlighted in Figure S1.

### Determination of the dye and protein concentration and degree of labeling (DOL)

The absorption spectra of each sample providing the absorbance values at the respective wavelength utilized for the determination of the dye and the protein concentration, i.e., the longest wavelength absorption maximum and 280 nm, were measured with a NanoPhotometer® NP80 from *Implen* (Munich, Germany). Equations (1)–(4) (see SI) were then used to determine the protein concentration and the DOL of antibody conjugates from these measurements. For all antibodies, a molecular mass of 150,000 g/mol and a molar extinction coefficient of 210,000 L mol^−1^ cm^−1^ were used. DOL determination of PEG-based intermediates was performed by analytical HPLC-SEC using the 1260 Infinity II LC System from *Agilent* and a flow rate of 0.35 mL/min. DOL values were then determined according to Eq. (5) (see SI). These DOL values present mean values due to the statistical distribution of the fluorophores per backbone. These numbers can be modified by adjusting the ratio of the molar equivalents of the monomeric dye or VB-dye intermediate to the respective antibody.

### Fluorescence spectroscopy and absolute determination of fluorescence quantum yields (QY)

QY of the monomeric and PEG-encapsulated dyes and their different conjugates were absolutely measured with a Quantaurus-QY Absolute PL quantum yield spectrometer from *Hamamatsu Photonics* (Shizuoka, Japan). This stand-alone integrating sphere setup is equipped with a 150 W xenon light source and automatic excitation wavelength control. Prior to the QY measurement, the sample concentration was set to an absorbance (OD) of 0.1 at the chosen excitation wavelength utilizing a spectrophotometer and 3.5 mL of the sample were transferred into a long-neck quartz cuvette. The *B* values of the monomeric dyes and the PEG-encapsulated multimeric fluorophores were calculated according to Eq. (6) (see SI).

### Fluorescence decay kinetics of the free and bead- or cell-bound samples and lifetime data

The fluorescence lifetimes of the various fluorophores in buffer solution were obtained on the fluorescence lifetime spectrometer FLS 920 from *Edinburgh Instruments* (Livingston, United Kingdom) with a R3809U-50 (range 200–850 nm, response width < 25 ps) from *Hamamatsu*, a multi-channel plate (MCP) detector, Czerny-Turner double monochromators utilizing an *Edinburgh Instrument* EPLED-485 (picosecond pulsed light emitting diode) for excitation at 485 nm. All measurements were performed in 10 mm quartz cuvettes from *Hellma GmbH* (Müllheim, Germany) filled with 2 mL of dye solution, diluted to an absorption value of 0.1 at the excitation wavelength. After each measurement, the instrument response function (IRF) was measured using a highly scattering colloidal solution (detection at the excitation wavelength). The lifetime data, i.e., the measured fluorescence decay curves, were analyzed with the FAST Software from *Edinburgh Instruments* and fitted with a reconvolution fit to the equation $I\left(t\right)=\sum_{i=1}^{n}{B}_{i}{e}^{-\frac{t}{{\tau }_{i}}}$. All lifetimes were evaluated mono or bi-exponentially.

FLIM imaging was performed with beads and SUP-T1 cells conjugated with different amounts of the assessed fluorescent labels. The samples were suspended in PBS buffer and transferred onto a coverslip. The fluorescence lifetime measurements were recorded with a FluoView FV1000 confocal microscope from *Olympus* (Tokyo, Japan) equipped with a FLIM fluorescence correlation spectroscopy upgrade kit from *PicoQuant* (Berlin, Germany). A diode laser with 485 nm and a repetition rate of 20 MHz, a dichroic mirror (DM405/488) and an *Olympus* objective UPLSAPO 60xW (numerical aperture 1.2 N.A.) and a Brightline FF01-520/35 bandpass filter were used for sample excitation and the detection of the subsequently emitted photons, using a single-photon avalanche diode (SPAD). Data acquisition and evaluation were conducted using the TimeHarp 200 TCSPC PC board and SymPhoTime software from *PicoQuant*. For the fitting of the measured fluorescence decay curves, the calculated IRF from the SymPhoTime software was used for a reconvolution fit with two or three lifetime components, employing the equation

$$I\left( t \right) = \mathop \sum \limits_{i = 1}^{n} B_{i} e^{{ - \frac{t}{{\tau_{i} }}}} .$$

### Confocal laser scanning fluorescence microscopy

Surface-stained cells (shown in Fig. 3 in the Results and Discussion section) were imaged on a Zeiss LSM 710 confocal microscope (Oberkochen, Germany; excitation with an Ar-laser at 488 nm, emission detected with 470/40 nm filter settings). The laser power was set to 5% and the gain to 675, respectively. Fluorescence intensities were quantified by integration of the signals derived from the fluorophore labeled membranes. The images were taken with a "Plan-Apochromat" 40x/0,95 Korr M27 objective and processed using Fiji software.

Stained cells and beads from Figs. S4 and S5 in the SI were imaged on a FluoView FV1000 *Olympus* (Tokyo, Japan) confocal laser scanning microscope. For sample excitation, a multiline argon ion laser (488 nm), was used. Excitation light was reflected by a dichroic mirror DM405/488 and focused onto the sample through an *Olympus* objective UPLSAPO 60xW (numerical aperture 1.2 N.A.). The emitted photons were recorded in the spectral window of 500–600 nm. The resulting images were processed using the ImageJ software.

## Results and discussion

### Probe design and bioconjugation

Fluorophore multimerization presents a simple and straightforward strategy to prepare brighter conjugates. However, for the majority of organic fluorophores, multimerization induces dye self-quenching and a label density-dependent QY for the resulting antibody-dye conjugates^20^. This encouraged us to explore PEG scaffolds to decrease unwanted dye-dye interactions often resulting in fluorescence quenching due to H-type dimer formation or homo-FRET^20–22^. With the aim to build up a platform for PEG-mediated multi-chromophoric labels containing a high number of unquenched fluorophores, we explored commercially available 20 kDa 8-arm PEG terminated with amino groups permitting the usage of well-established bioconjugation methods. To demonstrate the potential and applicability of this approach, we prepared a model system based on anti-CD4/CD8 antibodies and monomeric and multimeric dyes excitable at 488 nm. This wavelength presents the most frequently used (laser) excitation wavelength in flow cytometry and fluorescence microscopy. As representative fluorophores, the broadly applied dye fluorescein and the blue emitting dye Vio515, designed for applications in aqueous media, were selected. In the first step, NHS chemistry was used to obtain the VioBright (VB) dye intermediate, which was then chemically modified to an azide compound (see Fig. 1a, compound 2, and SI, Figure S2). The introduction of the azide group into the VB-dye intermediate allowed for the simple conjugation to DBCO-modified antibodies via a strain-promoted alkyne azide click (SPAAC) reaction^33^. Although this coupling strategy did not allow for the preparation of defined intermediates due to the statistical distribution of fluorophores and azide moieties on the PEG scaffold, however, represents a simple and straightforward method to obtain bright and conjugatable scaffolds.

Here, we used this method for conjugating the multimeric VB-dye label to the antibodies directed against CD4 and CD8 (Fig. 1b). To compare our conjugates, controls were prepared by the commonly applied direct lysine labeling of antibodies with the respective fluorophore NHS esters, providing statistical labeling and hence a distribution of fluorophore-biomolecule conjugates of varying degree of labeling. The application-relevant properties of fluorophores and their conjugates are summarized in Table 1.

**Table 1:** Overview of the spectroscopic properties of the monomeric fluorophores including the dyes utilized for antibody labeling, the PEGylated multimeric dyes, and the respective antibody-dye conjugates.

Fluorophores/labels	DOL	λ_abs_ (nm)	λ_em_ (nm)	QY	B (L mol^−1^ cm^−1^)
Fam NHS ester	−	495	514	0.49	34,000
Fam Na^+^ salt		496	516	0.88	62,000
Vio515 NHS ester	−	488	512	0.86	69,000
VioBright-Fam	5.3	496	523	0.78	289,000
VioBright-Vio515	5.4	489	511	0.83	359,000
CD4-Fam	13.8	494	519	0.18	174,000
CD4-Vio515	7.0	491	513	0.40	224,000
CD4-VioBright-Fam	14.0	497	522	0.70	686,000
CD4-VioBright-Vio515	13.0	489	512	0.81	842,000

The absorption and fluorescence spectra providing the spectral position of the respective maxima (λ_abs_; λ_em_) and the fluorescence quantum yields (QY) were measured in PBS (pH 7.4). The DOL equals the (average) degree of labeling of each conjugate. The brightness values were calculated according to Eq. (6) in the SI.

### Spectroscopic studies

**Figure 2:**
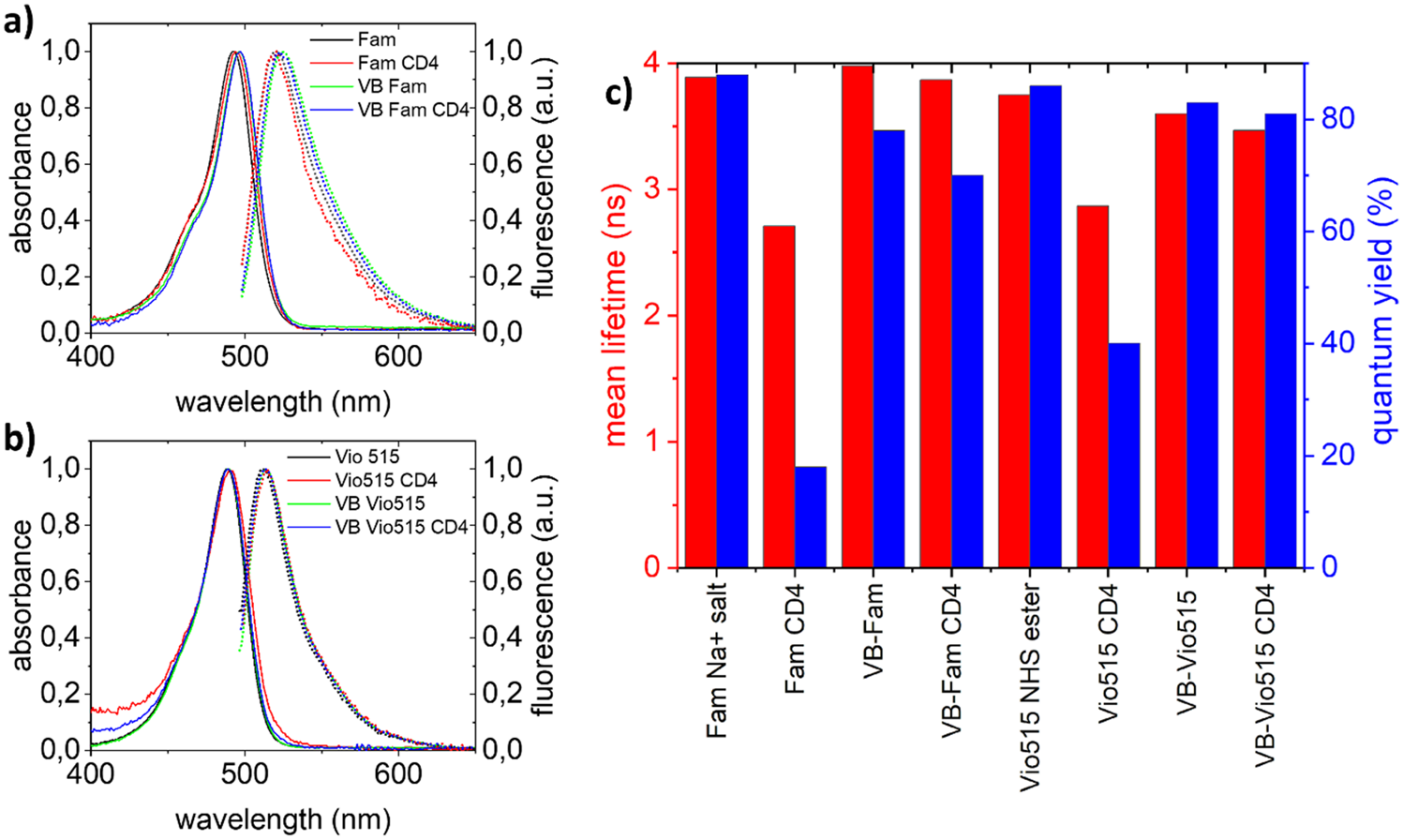
Spectroscopic characterization of the monomeric and multimeric Fam and Vio515 dyes and their CD4-antibody conjugates in PBS buffer. (**a**) and (**b**) Normalized absorbance and fluorescence spectra of monomeric and multimeric Fam and Vio515 and the respective CD4-conjugated labels. Excitation wavelength used for all fluorescence spectra: 488 nm. Solid lines: absorption spectra, dashed lines: fluorescence spectra. (**c**) Fluorescence quantum yields (blue) and intensity weighted mean fluorescence lifetimes (red) of the monomeric and multimeric Fam and Vio515 dyes and their CD4-antibody conjugates revealing a bioconjugation-induced drop in fluorescence lifetime and QY of the monomeric dyes and underlining the preservation of the fluorescence properties for the multimeric labels. Fam sodium salt was used as a reference since the 5/6-fluorescein NHS ester shows a strongly pH-dependent QY (see Table 1).

Absorption and fluorescence spectroscopic measurements of solutions of the monomeric and multimeric labels of the dyes Fam and Vio515 and the respective conjugates with the antibody directed against CD4 were performed in PBS buffer to explore the suitability of the chosen dye multimerization concept. Spectroscopic properties assessed included the spectral position and shape of the absorption and emission bands, signal-relevant photophysical quantities like QY, and the fluorescence decay kinetics providing the respective lifetimes. As shown in Fig. 2a and b, the spectral position and shape of the normalized absorption and emission spectra of monomeric Fam and Vio515 and their multimeric counterparts VioBright-Fam and VioBright-Vio515 closely match, underlining the absence of dye-dye interactions. The nearly complete preservation of the QY values and fluorescence decay kinetics and mean fluorescence lifetimes, highlighted in Fig. 2c, also demonstrate that encapsulation of Fam and Vio515 in these PEG structures clearly prevents dye-dye interactions. These findings confirm the excellent dye shielding provided by our multimerization concept.

Conjugation of the monomeric and multimeric labels with the antibodies targeting CD4 leads to a very small blue shift of the absorption and fluorescence spectra by 2 nm for the multimeric dye and its bioconjugates compared to the monomeric Fam dye and its bioconjugates. For the Vio515 dye, no spectral shifts are observed. As shown in Fig. 2c, bioconjugation of monomeric Fam and Vio515 results in a reduction in QY and mean fluorescence lifetime. Moreover, while the decay kinetics of the non-conjugated monomeric and multimeric labels are mono-exponential, bioconjugation leads to bi-exponential decay kinetics (see SI, Figure S7 and Table S8). The bioconjugation-induced decrease in QY observed for the monomeric Fam and Vio515 labels is attributed to dye self-quenching caused by dye-dye interactions between different CD4-bound monomeric dye molecules. Such dye-dye interactions commonly result in a diminution of QY as observed before for many fluorophores, e.g., for cyanine dyes^34^. While cyanine dyes have a considerable aggregation tendency in an aqueous environment, often resulting in the formation of non or barely emissive H-aggregates, which act as energy sinks for fluorescence quenching by homoFRET, fluorescein is less prone to dimerization and self-quenching. Nevertheless, a close proximity of the fluorescein molecules can still lead to self-quenching (see, e.g., Table 1 and Fig. 2c) via mechanisms that probably involve both static and dynamic quenching. Similar results were also reported by other groups. For example, Szabo et al., who examined the photophysical properties of the dyes AF546 and AF647 and their conjugates, observed a change in the absorption spectra and H-aggregate formation for the cyanine dye AF647, but not for the xanthene dye AF546. However, the fluorescence intensity of both fluorophores was reduced with increasing DOL^34^.

The fluorescence lifetime data of our samples provided in the SI suggest self-quenching of the dyes. (see Table S8). As depicted in Table S8, monomeric Fam and Vio515 fluorophores conjugated to CD4 antibodies show bi-exponential decay kinetics with a contribution of a second emissive species with a reduced fluorescence lifetime compared to that of the unbound parent dye, confirming a partial quenching of the fluorescence for the CD4 antibody conjugates of monomeric FAM and Vio515. An alternative explanation for the observed decay behavior could be a distribution of fluorescence lifetimes of the quenched species, which could be fitted by a sum of two exponential functions^35,36^. In contrast, in the case of the multimeric labels, bioconjugation induced only very small changes in QY for VB-Fam and did not affect the QY and the mean lifetimes/fluorescence decay kinetics of VB-Vio515. In both cases, the decay behavior of the multimeric labels remains mono-exponential, indicating that no quenching occurred. Overall, the spectroscopic properties of multimeric Fam and Vio515 and their bioconjugates underline the excellent shielding of the PEGylated dyes from each other, i.e., the prevention of dye-dye interactions, and suggest a protection from a potentially fluorescence quenching environment, at least for the bioconjugates.

### Functionality tests in flow cytometry and confocal microscopy

**Figure 3:**
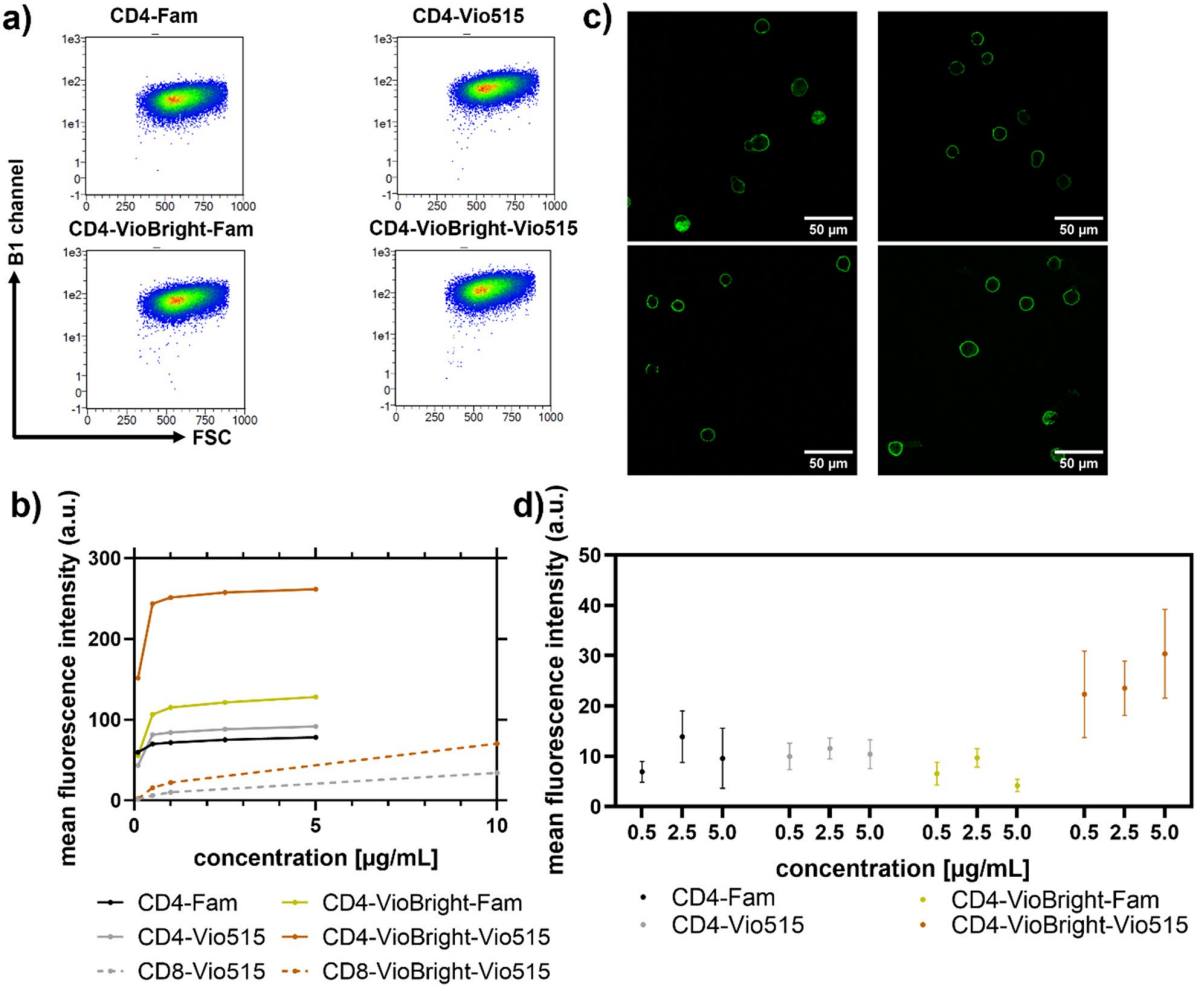
Quantification and comparison of the mean fluorescence intensities (MFI) of the different monomeric and multimeric labels used for the staining of the SUP-T1 cells. (**a**) Representative flow cytometry dot plots obtained for fixed SUP-T1 cells stained with CD4 conjugates at 10 µg/mL. The y-axis represents the mean fluorescent intensity detected in the B1 channel and the x-axis the forward scatter. (**b**) Quantification of the fluorescent signals of each conjugate measured in different concentrations on fixed SUP-T1 cells using flow cytometry. In cell staining experiments, the multimeric VioBright dye conjugates (yellow and orange lines) provide a higher fluorescent signal in comparison to their monomeric dye conjugates (black and grey lines). (**c**) Representative CLSM images of the CD4 conjugates utilized for cell staining at 5 µg/mL targeting the surface receptor CD4 & CD8 of fixed cells and (**d**) quantification of surface staining at the given conjugate concentrations. The cells were incubated with conjugates for 10 min at room temperature. The scale bar equals 50 µm.

The brightness and functionality of our conjugates were examined with flow cytometry and confocal laser scanning microscopy (CLSM). As a model system for the staining with the antibody conjugates of our monomeric and multimeric labels, we chose the T cell lymphoma cell line SUP-T1 which expresses both CD4 and CD8 receptors (see Fig. 3a and b). The flow cytometry data confirm that VioBright-mediated fluorophore multimerization leads to increased mean fluorescence intensities (MFI) compared to their monomeric counterparts. Furthermore, the obtained MFI with the CD4 conjugates did not further increase with concentrations higher than 1 µg/mL, whereas CD8-targeted conjugates showed a steady increase in fluorescence signal intensity up to a concentration of 10 µg/mL due to decreased affinity of the anti-CD8 antibody to already fixed epitopes. This could be omitted in the future by staining prior to fixation as recommended by the manufacturer. To test the functionality of the dye-conjugated antibodies exploited for the staining of the CD4 receptors, i.e., their target binding, we performed CLSM studies. These experiments revealed selective membrane staining (Fig. 3c). Figures S4 and S5 in the SI show representative results using the CD4-conjugated Fam dye bound to beads and SUP-T1 cells obtained for different labeling concentrations. Cells and beads treated with high label concentrations showed higher fluorescence intensities than lower labeling concentrations. The same behavior was observed for all dyes and labels on beads and on cells. Moreover, for the VioBright-Fam conjugates under CLSM conditions, we observed a more pronounced photobleaching (Fig. 3d, see also SI, Figure S6) resulting in lower staining intensities. Nevertheless, the functionality of the VioBright-derived conjugates and their counterparts could be demonstrated. When comparing only VioBright and only monomeric species (Fig. 3a and b), the observed fluorescence intensities are in good agreement with the previously determined B values.

### Fluorescence lifetime imaging studies

In addition to brightness and photostability, many applications of flow cytometry and fluorescence microscopy require the simultaneous detection and discrimination of different target structures like various receptors of cancer cells or rare cells in a sample with the limited number of fluorescence detection channels^37^. Parameters exploited for target or analyte detection and multiplexing and barcoding schemes are presently mainly fluorescence intensity and emission color/spectrum^38^. In flow cytometry, the achievable degree of spectral color multiplexing is determined by the size of the label´s Stokes shift and the spectral width of its emission bands, that jointly control spectral cross talk in excitation and emission^39^. Approaches proposed to increase the degree of spectral multiplexing include high-throughput imaging with releasable antibodies^40^, co-detection by indexing^41^, and releasable fluorophores^42^. Also the label- and microenvironment-specific, but concentration-independent parameter fluorescence lifetime can be exploited which can be derived from the label´s fluorescence decay kinetics accessible with time-resolved spectroscopic or microscopic techniques^43^. This parameter has been previously mainly utilized for time gating with fluorescent labels with long lifetimes such as lanthanide complexes or chelates and energy transfer systems using lanthanide complexes or Mn(II)-doped quantum dots as donors for an efficient background suppression^38,44–46^. Recent examples present imaging applications utilizing fluorescence lifetime imaging (FLIM) and lifetime multiplexing in conjunction with different types of fluorophores and different fluorescence techniques^4,47–56^. Meanwhile, the potential of the parameter fluorescence lifetime has been also recognized for analyte discrimination and multiplexing. This concept, which can be also combined with spectrally resolved measurements, is currently explored for different types of fluorophores^57–60^. For flow cytometry, which relies on fluorescence measurements in a flow with a very short time window of interaction between the label and the excitation light and the collection of a relatively small number of photons, bright molecular labels with a ns lifetime are preferred^55,61,62^.

Subsequently, to further explore the application potential of our multimeric labels, we explored the applicability of the multimeric dyes for lifetime discrimination and multiplexing in conjunction with FLIM in a proof-of-concept experiment. Therefore, we assessed a possible discrimination of epitopes stained with antibody conjugates of our monomeric and multimeric fluorophores for cell samples bearing CD4 and CD8 proteins by means of fluorescence lifetime measurements and FLIM. These receptors are usually expressed in certain T cell subsets and thereof derived cell lines (e.g., SUP-T1). As we had observed more pronounced photobleaching for the VioBright-Fam conjugates under CLSM conditions (see SI, Figure S6), resulting in lower staining intensities as described in the previous section, for the FLIM studies, we thus employed controls that allow for a morphological discrimination when imaged in the same spectral channel. For the preparation of the controls, we incubated our conjugates with anti-REA MACS® Comp beads, which bind to the constant region of every antibody and are distinguishable from cells by their spherical shape and size of approximately 5 µm.

### FLIM studies of beads and cells stained with CD4-dye conjugates

**Figure 4:**
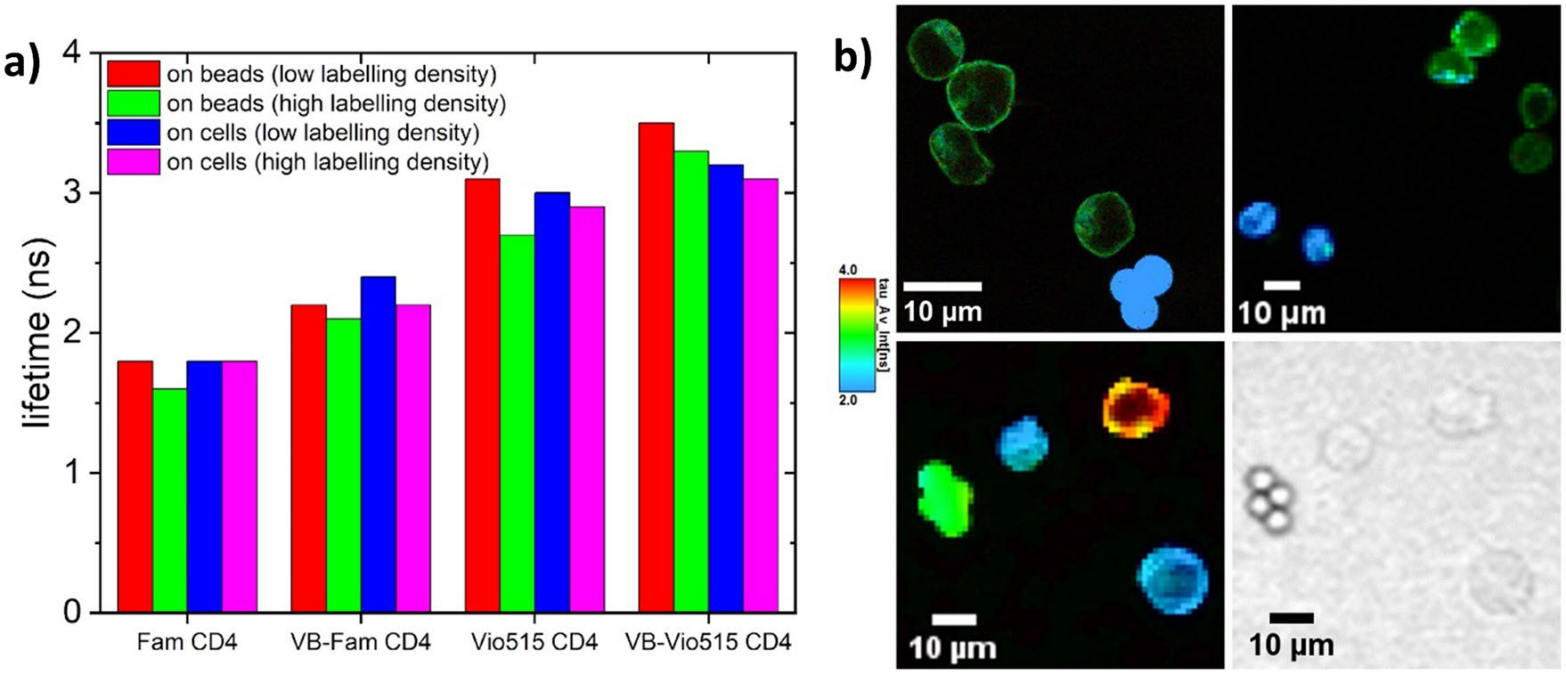
(**a**) Intensity weighted mean lifetimes of the CD4-conjugated dyes and labels bound to beads and SUP-T1 cells in low and high labeling densities, as calculated from the FLIM measurements (for more details see SI). (**b**) Representative FLIM measurement results demonstrating the principal feasibility of the reported dyes and labels for lifetime-based analyte discrimination. Top left: Mixture of beads stained with CD4-Fam (low labeling density) and SUP-T1 cells with CD4-VB-Fam (high labeling density). Top right: Mixture of SUP-T1 cells with CD4-Fam and CD8-Vio515 (both with high labeling density). Bottom left: Mixture of SUP-T1 cells with CD4-Fam and with CD4-VB-Vio515 (both with high labeling density) and beads with CD4-VB-Fam (low labeling density). Bottom right: Transmission image for morphological identification in the bottom left image.

FLIM measurements of cells and beads carrying the CD4 target and stained with the respective CD4-dye conjugates were performed in PBS buffer. The results are summarized in Fig. 4a, showing the intensity weighted mean lifetimes for each sample calculated from the FLIM measurements. For each bead and cell sample, three individual FLIM images were evaluated and utilized for the calculation of the average fluorescence lifetimes in Fig. 4a. The measured intensity weighted mean fluorescence lifetimes slightly differ between the monomeric and multimeric labels and are barely affected by labeling density as revealed by the similar mean fluorescence lifetimes obtained for the CD4-bearing beads or cells with low and high labeling densities. The differences between the mean lifetimes obtained for the bioconjugates of the monomeric and multimeric reporters in PBS buffer shown in Fig. 2c and the mean lifetimes of the respective fluorescent bioconjugates bound to cells and beads are ascribed to partial fluorescence quenching caused by the microenvironment in the close proximity of the CD4-dye conjugates. Although our PEGylation-based multimerization concept provides an excellent suppression of dye-dye interactions, as demonstrated by the results of our spectroscopic studies, it cannot completely prevent through space quenching by the reporter microenvironment. For example, in the case of fluorescein, fluorescence quenching by dye environment, e.g., by tryptophane in the dye´s neighborhood has been reported^63^.

However, as revealed by the comparison of the mean fluorescence lifetimes of the target-bound bioconjugates of the monomeric and multimeric labels on beads and cells, our multimerization concept nevertheless reduces such detrimental environmental effects on reporter fluorescence. Possibly, the higher power density in a confocal microscopy setup could possibly lead to a faster photodecomposition of the bead- and cell-bound labels than observed for their free counterparts in solution, the fluorescence lifetimes of which were recorded with a fluorescence lifetime spectrometer at a considerably lower excitation power density. This assumption is in good agreement with the higher photostability of the Vio515-based labels compared to the Fam-based labels (see SI, Figure S6). Additional differences in data evaluation may arise from the calculated IRF used in the FLIM-measurements of bead- and cell-bound labels compared to the IRF recorded for each individual measurement in the fluorescence lifetime spectrometer for the solutions of the respective free labels.

### Lifetime multiplexing using FLIM

To explore the potential of our monomeric and multimeric labels for lifetime-based analyte discrimination, we performed FLIM measurements with mixtures of samples containing beads and SUP-T1 cells labelled with different anti-CD4 conjugates (Fig. 4b). Figure 4b (top left image) shows a comparison of the FLIM results obtained with monomeric and multimeric Fam labels bound to the CD4-target on beads and on SUP-T1 cells. This figure demonstrates the possibility to distinguish between both species based upon lifetime measurements. In the bottom left image of Fig. 4b, results of FLIM measurements obtained with a mixture of three different species, i.e., CD4-Fam and CD4-VB-Vio515 bioconjugates bound to cells (both with high labeling density) and CD4-VB-Fam bound to beads (low labeling density). The difference in labeling density between beads and cells is due to the different conjugate concentrations (see Fig. 3b) of the two carrier systems (monomeric vs. PEG-based) on beads or cells used in this study. In this experiment, the bioconjugates with the shortest and longest mean lifetimes of 1.8 ns and 3.1 ns were utilized for staining of CD4 epitopes on cells, whereas the dye-bioconjugate with a medium mean lifetime of 2.2 ns was used for the labeling of proteins bound to beads, which recognize Fc parts of our fluorescent labels. This clearly demonstrates that the three different species can be distinguished by FLIM measurements using the same excitation and emission wavelengths based on their fluorescent lifetimes. As follows from the bottom right image of Fig. 4b, the results of the FLIM-based discrimination of the different cells and beads can be confirmed by morphological (e.g. shape and size) analysis.

## Conclusion and outlook

In this study we performed systematic spectroscopy studies of the multimeric dyes Fam and Vio515 and their monomeric parent fluorophores conjugated to anti-CD4 antibodies. Our results confirm that our versatile dye multimerization concept, that relies on simple PEG scaffolds, yields bright and bioconjugatable multimeric labels consisting of non-quenched dye molecules. As revealed by the matching absorption and emission spectra, fluorescence quantum yields, and fluorescence decay kinetics/fluorescence lifetimes of the monomeric and multimeric dyes, this multimerization strategy can suppress dye-dye interactions resulting in the formation of H-type dimers and fluorescence quenching by energy transfer processes between aggregated non- or barely emissive fluorophores and monomeric fluorescent molecules. Moreover, conjugation of the multimeric dyes Fam and Vio515 to the antibody directed against CD4 did not affect the signal-relevant absorption and fluorescence properties of both reporters, while the monomeric dyes revealed a reduction in fluorescence quantum yield and lifetime. This suggests also at least a partial protection of the PEGylated dyes from possible quenchers in their immediate environment. Overall, with the aid of our simple PEG-shielding scaffold, brighter labels and bioconjugates and an increase in label brightness by a factor of about three could be realized compared to conventional dyes and dye bioconjugates with comparable labeling densities could be prepared.

As revealed by microscopy and fluorescence lifetime imaging (FLIM) studies of CD4-dye conjugates of the monomeric and multimeric dyes Fam and Vio515, all conjugates were well suited for the staining of cells and beads bearing the respective target or antigen. Although the FLIM data of the stained beads and cells revealed a slight reduction in mean fluorescence lifetime also for the multimeric labels compared to the lifetimes measured by time-resolved fluorescence spectroscopy in PBS buffer, these effects were less pronounced as observed for the monomeric dyes, particularly in the case of Vio515. Apparently, the multimerization concept cannot provide a complete shielding of the encapsulated fluorophores from through space fluorescence quenching. In the case of pH-sensitive Fam, these findings may point to pH changes in the immediate dye environment. These changes in fluorescence lifetime of the different bioconjugates could be exploited for the lifetime-based discrimination of different, yet morphologically identical cell populations and bead populations. Based on our FLIM measurements and the different mean fluorescence lifetimes, three species could be distinguished using the same excitation and emission wavelengths. This could be, e.g., utilized in the future for lifetime multiplexing applications in FLIM.

In the future we will explore whether we can further boost reporter and conjugate brightness by increasing the degree of dye multimerization. This could be particularly promising for applications that require the detection of low-expressed targets and speed up signal detection. In addition, we will extend our multimerization concept to other fluorophores, especially to near infrared emissive dyes, that suffer from relatively low fluorescence quantum yields compared to fluorophores with emission in the visible and ultraviolet region. In the future, we plan to develop platforms of multimeric vis/NIR labels with reactive groups such as azide or thiol functionalities which can be excited with laser sources commonly used in fluorescence microscopy and flow cytometry. Moreover, we will explore the potential of our multimeric labels for lifetime multiplexing. This can eventually increase the number of simultaneously measurable targets for fluorophores with closely matching absorption and emission features and multi-parameter imaging and flow analysis of complex samples.

### Supplementary Information

Supplementary Information.

## Data Availability

All data generated/analyzed during this study are included either in this article and its Supplementary Information files or are available upon request to the corresponding author (D. A. Yushchenko, dmytroy@miltenyi.com; U. Resch-Genger, ute.resch@bam.de) or the first author (T. Reiber, thorger@miltenyi.com).
