# Supplementary material for: Fluorophore multimerization on a PEG backbone as a concept for signal amplification and lifetime modulation
Source: Sci Rep. 2024 May 24;14:11882. doi: 10.1038/s41598-024-62548-4 (PMC11126734; doi:10.1038/s41598-024-62548-4)
Supplement: Supplementary file 1 — Supplementary Information. [file 41598_2024_62548_MOESM1_ESM.pdf]

Supplementary Information for:

# Fluorophore multimerization on a PEG backbone as a concept for signal amplification and lifetime modulation

Thorge Reiber[1,3,#], Oskar Hübner[2,#], Christian Dose[1], Dmytro A. Yushchenko[1,*], and Ute Resch-Genger[2,*]

[1]Department of Chemical Biology, Miltenyi Biotec B.V. & Co. KG, Friedrich-Ebert-Straße 68, 51429 Bergisch Gladbach, 51429, Germany

[2]Division Biophotonics, Federal Institute for Materials Research and Testing (BAM), Richard-Willstaetter-Str. 11, 12489 Berlin, Germany.

[3]Department of Chemistry, Humboldt-Universität zu Berlin, Brook-Taylor-Str. 2, 12489 Berlin, Germany

[#]these co-first authors contributed equally to this work

[*]corresponding authors, email: dmytroy@miltenyi.com; ute.resch@bam.de

**Contents**

1. Reagents and chemicals ..... 2
2. Purchased binders, conjugates, PEGs, and beads ..... 2
3. Cell culture ..... 2
4. Staining procedure ..... 3
5. Flow cytometry ..... 3
6. Conjugation Procedures ..... 4
7. Determination of dye and protein concentration and degree of labeling (DOL) ..... 5
8. Analytical High-Performance Liquid Chromatography-Size Exclusion Chromatography (Analytical HPLC-SEC) ..... 6
9. Fluorescence Quantum Yield measurements ..... 7
10. Brightness ..... 8
11. Product & intermediate purification ..... 8
12. Confocal laser scanning microscopy (CLSM) ..... 10
13. Photostability ..... 11
14. Decay behavior of free labels in PBS buffer solution ..... 12
15. References ..... 13

## 1. Reagents and chemicals

All recombinant antibodies and Vio515 NHS ester were produced by *Miltenyi Biotec* (Bergisch Gladbach, Germany). 5/6-Carboxyfluorescein NHS ester and fluorescein sodium salt were purchased from *Thermo Fisher* (Waltham, MA, USA). 20 kDa 8-arm PEG Amine (hexaglycerol core) HCl salt was purchased from *JenKem* (Beijing, China). Azidoacetic acid NHS ester and DBCO-PEG$_4$ NHS ester were purchased from *Jena Bioscience* (Jena, Germany). All reagents were used without further purification or analysis. Phosphate buffered saline (PBS) pH 7.4 was produced by *Miltenyi Biotec*. Carbonate buffer pH 8.3 and 500 mM carbonate buffer pH 9.0 were prepared once by using desalted and sterile-filtered water. PEB buffer was prepared with PBS pH 7.4, 2mM EDTA and 0.5% bovine serum albumin. Dimethyl sulfoxide was purchased from *Thermo Fisher*.

## 2. Purchased binders, conjugates, PEGs, and beads

**Table S1**. List of antibodies, conjugates, beads, and PEG used in this study.

| **Antibodies** | **Purchased from** |
|---|---|
| CD4 antibody, anti-human, pure, REAfinity™ | Miltenyi Biotec |
| CD8 antibody, anti-human, pure, REAfinity™ | Miltenyi Biotec |
| **Beads** | |
| MACS® Comp Bead Kits, anti-REA | Miltenyi Biotec |
| **Conjugates** | |
| CD4 VioBright B515 antibody, anti-human, REAfinity™ | Miltenyi Biotec |
| CD4 VioBright FITC antibody, anti-human, REAfinity™ | Miltenyi Biotec |
| **PEG** | |
| HG 8-arm-PEG (8 $NH_2$), M = 20,000 g/mol | JenKem |

## 3. Cell culture

SUP-T1 cells were cultivated in Roswell Park Memorial Institute (RPMI 1640) medium from *Biowest* (Nuaillé, France) supplemented with 10% fetal calf serum (FCS) from *Biochrom* (Berlin, Germany) and 2 mM L-glutamine from *Lonza* (Basel, Switzerland). The cells were maintained at 37°C and 5% $CO_2$.

## 4. Staining procedure

Cells were fixed for 20 min in 3.7% formaldehyde solution available from *Miltenyi Biotec* (Bergisch Gladbach, Germany) at room temperature (RT). PFA was removed and cells were washed one time with PEB buffer for 5 min and stored at 4 °C prior to staining. Bead surface stainings were performed in the same manner as cell stainings, however without formaldehyde treatment and directly in the buffer containing the beads. Antibody-dye conjugates were adjusted to 50 µg/ml in PBS and incubated with cells/beads for 10 min at RT in the dark resulting in final conjugate concentrations between 0.1 and 10 µg/mL (also referred as low and high labeling densities). Cells/beads were centrifuged (2000 rpm, 5 min), staining solution was removed, and the cells/beads were resuspended in PEB buffer. Staining was performed in Eppendorf tubes and the resulting samples were pipetted into either 96-well plate for flow cytometry analysis or 24-well glass plate for confocal microscopy.

## 5. Flow cytometry

Flow cytometry data were obtained with a MACSQuant X Analyzer from *Miltenyi Biotec* with 488 nm laser excitation, emission detection in B1 channel and medium flow rate. 400,000 cells/well in a 96 well-plate were stained and 50.000 events recorded at medium flow rate. Data analysis was carried out using MACSQuantify software version 2.13.0. The general gating strategy was 1) to select the main population followed by 2) exclusion of doublets/aggregates and lastly 3) plot the B1 signal against the forwards scatter signal (see Figure S1). Fluorescence intensities were obtained as mean fluorescence intensity (MFI) values of the whole population in the B1 channel.

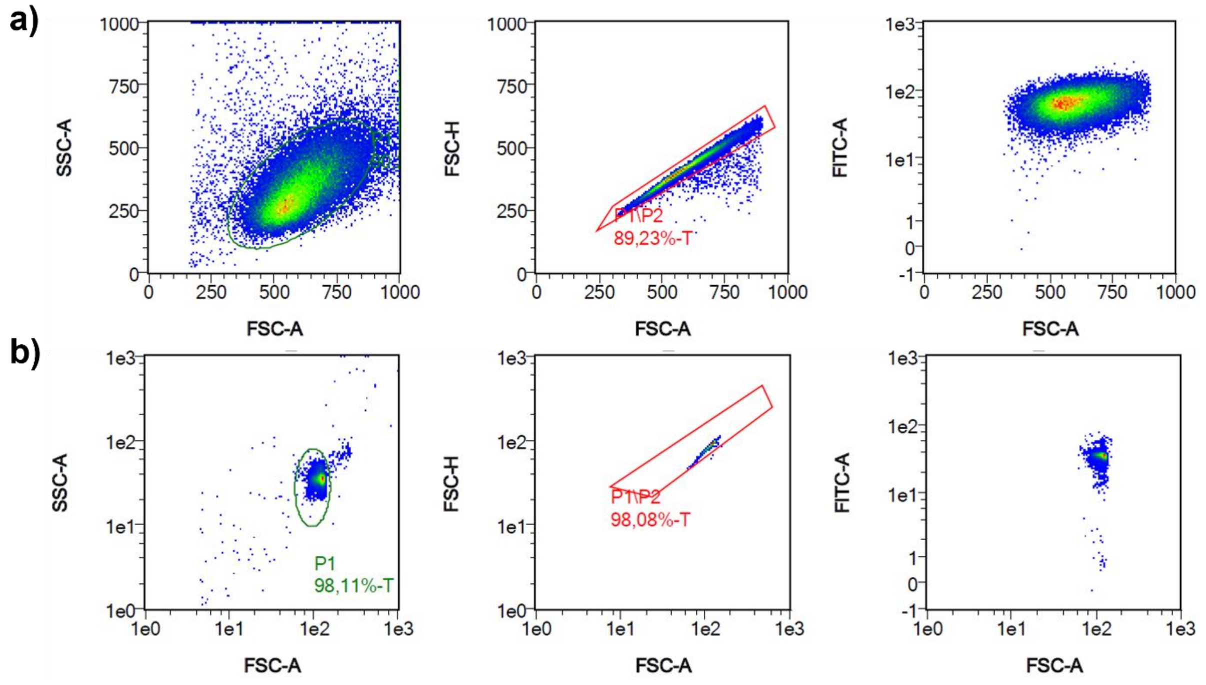


**Figure S1**. a) Gating strategy for stained SUP-T1 cells and b) beads. For both, the main population was selected and doublets excluded. Lastly, forward scatter was plotted against B1 (Fam) channel.

## 6. Conjugation Procedures

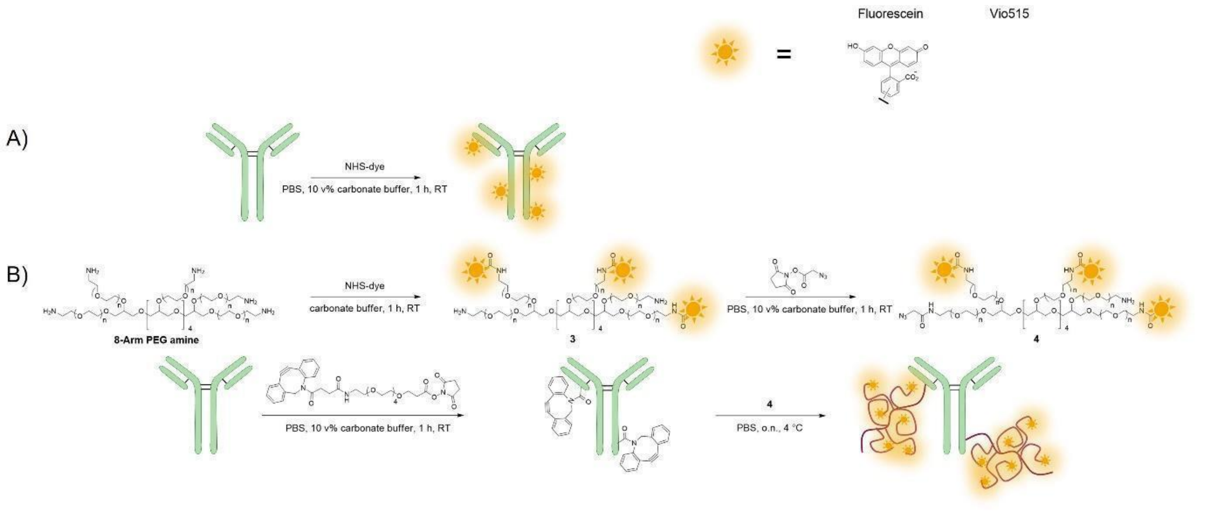


**Figure S2**. Reaction scheme towards (**A**) conventional antibody-dye conjugates *via* activated NHS esters of the respective fluorophore. (**B**) depicts the route to obtain antibody-VB-dye conjugates *via* SPAAC click reaction with **4** as intermediate.

**NHS ester antibody conjugation.** For each antibody fluorophore conjugation, NHS ester of the respective dyes dissolved in DMSO was added in a ratio of 10:1 under vortexing to a solution of antibody in PBS pH 7.4 with 10 vol% carbonate buffer pH 9.0 and left on the shaker for 1 h in the dark at room temperature. The final antibody concentration during conjugation was set to 4.5 mg/mL. To prevent protein degradation, DMSO content was kept below 5 vol%. The reaction mixture was then directly applied onto a NAP column for purification.

**VioBright dye conjugation.** For each VioBright fluorophore conjugation, NHS ester of the respective dyes dissolved in DMSO was added in a ratio of 8:1 under vortexing to a solution of polymer in carbonate buffer pH 8.3 and left on the shaker for 1 h in the dark at room temperature. The final polymer concentration during conjugation was set to 10 mg/mL. The reaction mixture was then directly applied onto a NAP column for purification.

**Activation of antibodies.** Antibodies (final concentration 4.5 mg/mL) were activated with DBCO-$PEG_4$ NHS ester dissolved in DMSO (10 mg/mL) in a molar ratio of 8:1 (DBCO compound to antibody) for 1 h in the dark in PBS buffer pH 7.4 containing 10 vol% carbonate buffer pH 9.0. The reaction mixture was then directly applied onto a NAP column for purification. Protein-containing fractions were determined *via* Commassie reagent and pooled accordingly in Eppendorf tubes.

**Activation of VioBright-dye intermediates.** In order to couple the VB-dye intermediates to antibodies, they were activated with azidoacetic NHS ester. For this purpose, $N_3$Ac-NHS was dissolved in DMSO (10 mg/mL) and added to a solution of polymer-dye in PBS pH 7.4 with 10 vol% carbonate buffer pH 9.0 in a ratio of 10:1 under vortexing. The final VB-dye concentration during conjugation was set to 2.5

mg/mL. The reaction mixture was left on the shaker for 1 h in the dark prior to purification *via* NAP column.

**Antibody polymer-PEG-dye conjugation.** Azido-modified VB-dye was added to the DBCO-activated antibody in a 7.5-fold molar excess in PBS pH 7.4 at a final antibody concentration of 5 mg/mL and left in the fridge at 4 °C overnight in the dark. The reaction mixture was then directly transferred to the ÄKTA system for purification.

## 7. Determination of dye and protein concentration and degree of labeling (DOL)

Protein and fluorophore concentrations as well as the degree of labeling (DOL) were determined from absorption measurements utilizing the absorbance at 280 nm for the determination of the protein concentration and the respective absorbance at the longest wavelength absorption maximum of each dye. These measurements were done with the photometer NanoPhotometer® NP80 from *Implen* (Munich, Germany). The following equations were subsequently used:

$$c_{protein} = \frac{OD_{280}^{corr} \cdot d}{\varepsilon_{protein}} \qquad (1)$$

$$c_{dye} = \frac{OD_{dye} \cdot d}{\varepsilon_{dye}} \qquad (2)$$

$$OD_{280}^{corr} = OD_{280} - (OD_{dye} \cdot cf) \qquad (3)$$

$$DOL = \frac{c_{dye}}{c_{protein}} \qquad (4)$$

c: concentration of compound in mol/L

OD: absorbance at the respective wavelength

d: dilution factor

ε: molar extinction coefficient of protein or fluorophore in L/mol at their respective wavelength

cf: correction factor of the dye at 280 nm

For all antibodies, a molecular mass of 150,000 g/mol and an extinction coefficient of 210,000 $Lmol^{-1}cm^{-1}$ was used.

## 8. Analytical High-Performance Liquid Chromatography-Size Exclusion Chromatography (Analytical HPLC-SEC)

The DOLs of VB-dye conjugates were analyzed by HPLC-SEC using the 1260 Infinity II LC System from *Agilent*. For SEC, the AdvancedBio SEC 2.7 µm column (4.6 x 300 mm) and Agilent AdvancedBio SEC 2.7 µM (4.6 x 50 mm) as guard column were used. PBS (pH 7.4) was used as mobile phase. The conjugates were dissolved in PBS. The parameters for the method are given below.

<u>Analytical method:</u>

Flow rate: 0.350 ml/min; UV-Detection: 489 nm (Vio515) and 495 nm (Fam).The DOL of the fluorophore-VioBright intermediate was calculated from the area under the curve (AUC) of reacted and unreacted fluorophores as well as molar ratio:

$$DOL = \frac{AUC_{VB-dye}}{AUC_{VB-dye} + AUC_{free\ dye}} \cdot ß \quad (5)$$

ß: used molar ratio of fluorophore to VioBright

AUC: area under the curve

<u>VB-Fam</u> (8.7 min)

aggregates (5.4, 7.5 min), free Fam (12.1 min)

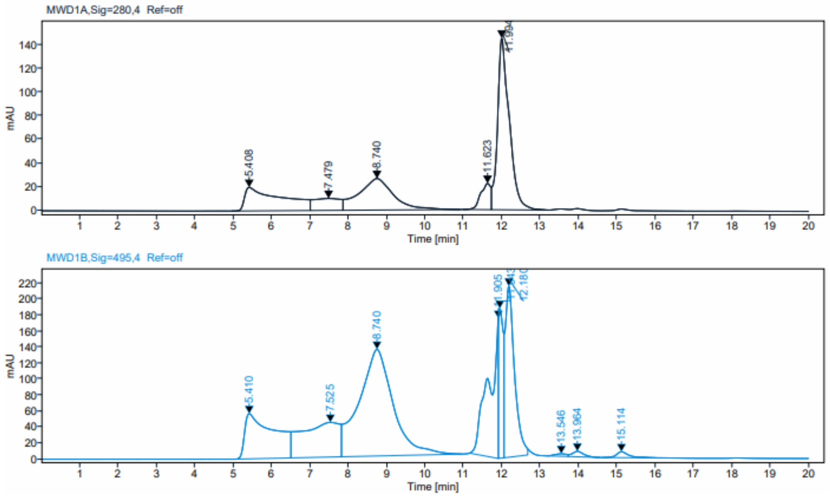

VB-Vio515 (8.6 min)

aggregates (7.4 min), free Vio515 (11.6 min)

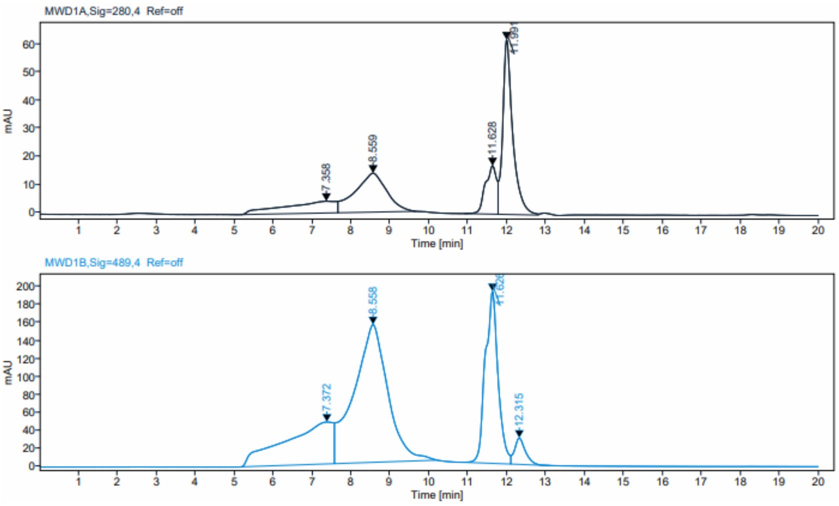


| **Labels** | **DOL** | **$AUC_{bound}$** | **$AUC_{total}$** | **ß** |
|---|---|---|---|---|
| VB-Fam | 5.3 | 15,085 | 22,666 | 8 |
| VB-Vio515 | 5.4 | 12,294 | 18,231 | 8 |

## 9. Fluorescence Quantum Yield measurements

The fluorescence quantum yields of the monomeric and PEG-encapsulated dyes and their different conjugates were measured with a Quantaurus-QY Absolute PL quantum yield spectrometer from *Hamamatsu Photonics* (Shizuoka, Japan). This stand-alone integrating sphere setup is equipped with a 150 W xenon light source and automatic excitation wavelength control. Prior to the measurement, the sample concentration was set to an absorbance of 0.1 OD with a spectrophotometer. 3.5 mL of the respective sample solution was then transferred into a long-neck quartz cuvette. Excitation done at the sample´s absorption maximum. As blanks, the respective non-emissive solvents were used. The fluorescence quantum yield, equaling the number of emitted per number of absorbed photons, was determined by integration of the spectrally corrected emission spectra after subtraction of the blank signal while the number of absorbed photons was derived from the chance in the intensity of the incident excitation light detected for the blank, attenuated by sample absorption at the excitation wavelength. All measurements were done in triplicates if not stated otherwise to derive relative standard deviations.

## 10. Brightness

The brightness (B) of the monomeric dyes and the PEG-encapsulated multimeric fluorophores was calculated according to the following equation:

$$B = DOL \cdot \varepsilon \cdot QY \qquad (6)$$

ε : molar extinction coefficient at the chosen excitation wavelength in [$LMol^{-1}cm^{-1}$]. Excitation wavelength was chosen based on the absorption maximum.

QY: fluorescence quantum yield

Although the signal size directly correlates only with the brightness value determined at the respective excitation wavelength[1],we always used the wavelength of the respective absorption maximum for the calculation of the B values, as we aimed only for a comparison of the monomeric and multimeric Fam and Vio515 fluorophores with closely matching absorption spectra.

## 11. Product & intermediate purification

Obtained intermediates as well as antibody-dye conjugates were purified using NAP columns from *Cytiva Lifescience* (Marlborough, MA, USA) pre-packed with Sephadex G-25 Grade DNA resin. For this purpose, the columns were equilibrated with degassed PBS pH 7.4 and the sample eluted according to the procedure of the manufacturer. Product-containing fractions were then pooled in an Eppendorf tube.

Final conjugates were purified on an ÄKTA pure 25 chromatography system equipped with a Superdex 200 Increase 10/300 GL column from *Cytiva Lifesciences*. Elution was done with PBS pH 7.4 at a flow rate of 0.75 mL/min and fraction size of 0.5 mL. Detection was achieved by absorbance at 280 nm (antibodies) and 495 nm (FAM, Vio515). Fractions corresponding to the desired product were collected, pooled, and concentrated via Amicon filtration to an appropriate volume.

a)

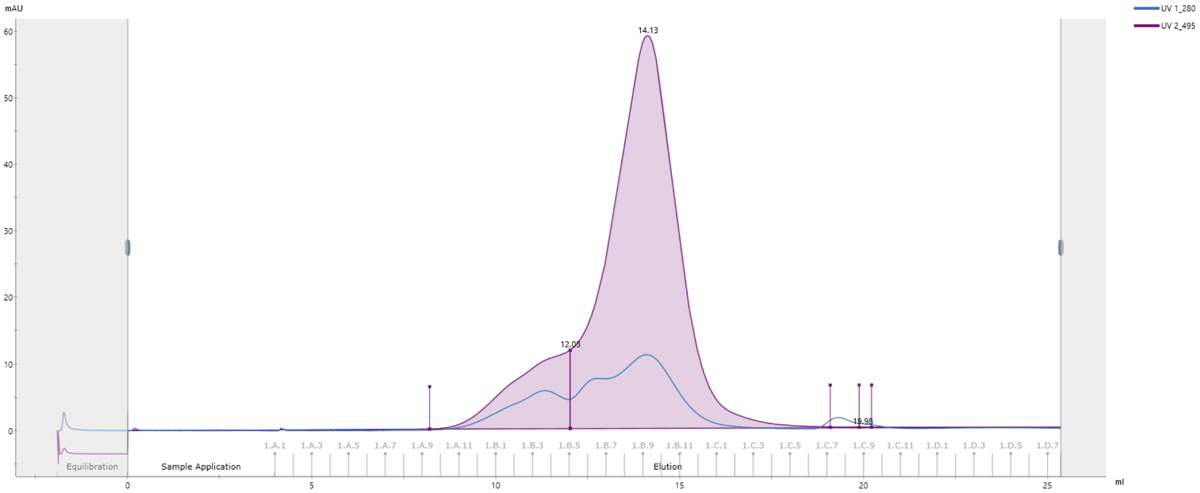
mAU
UV 1_280
UV 2_495
14.13
12.03
19.98
Equilibration
Sample Application
Elution
ml

b)

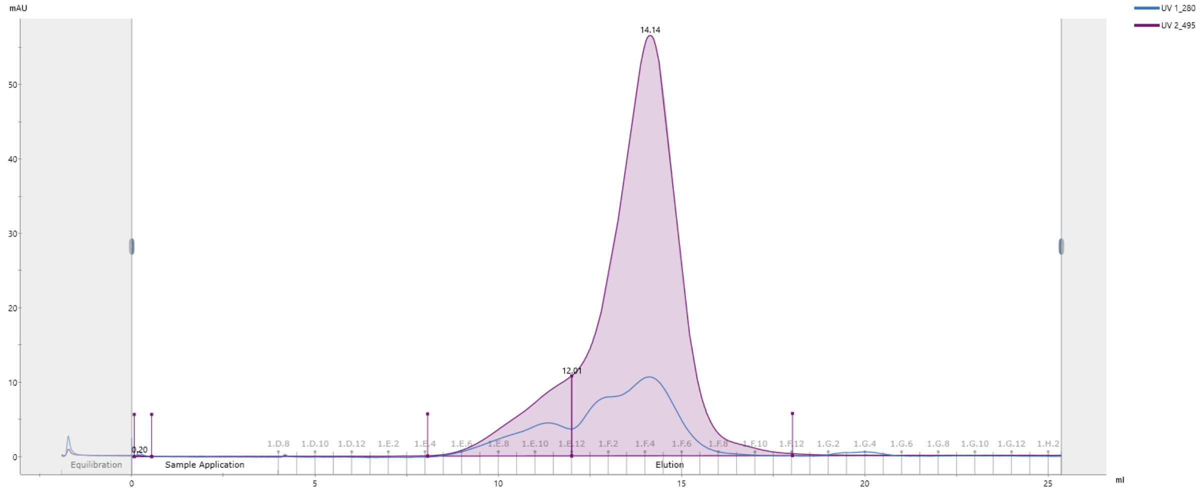
mAU
UV 1_280
UV 2_495
14.14
12.01
0.20
Equilibration
Sample Application
Elution
ml

c)

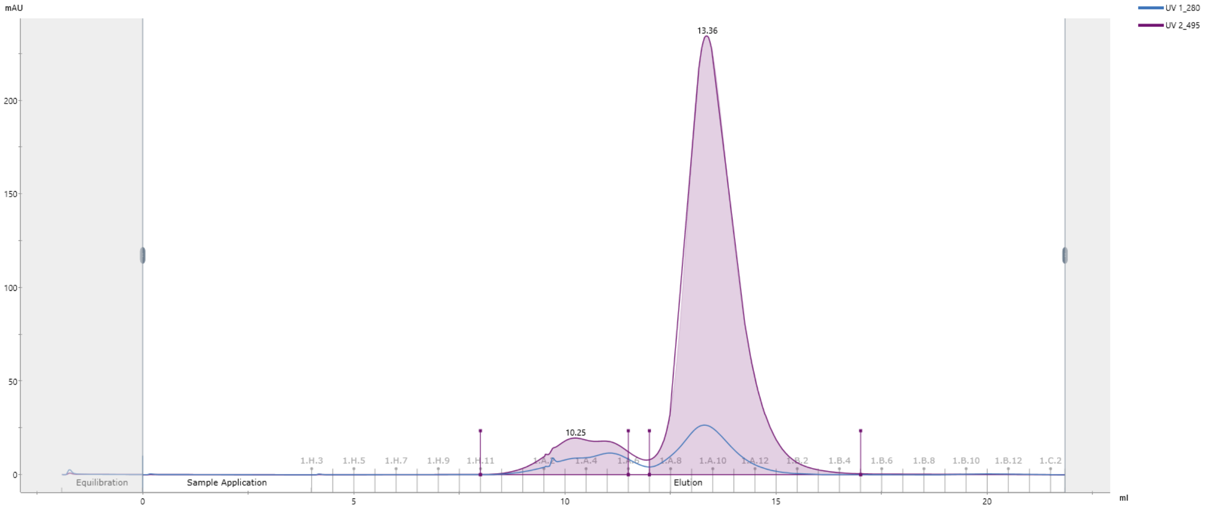
mAU
UV 1_280
UV 2_495
13.36
10.25
Equilibration
Sample Application
Elution
ml

d)

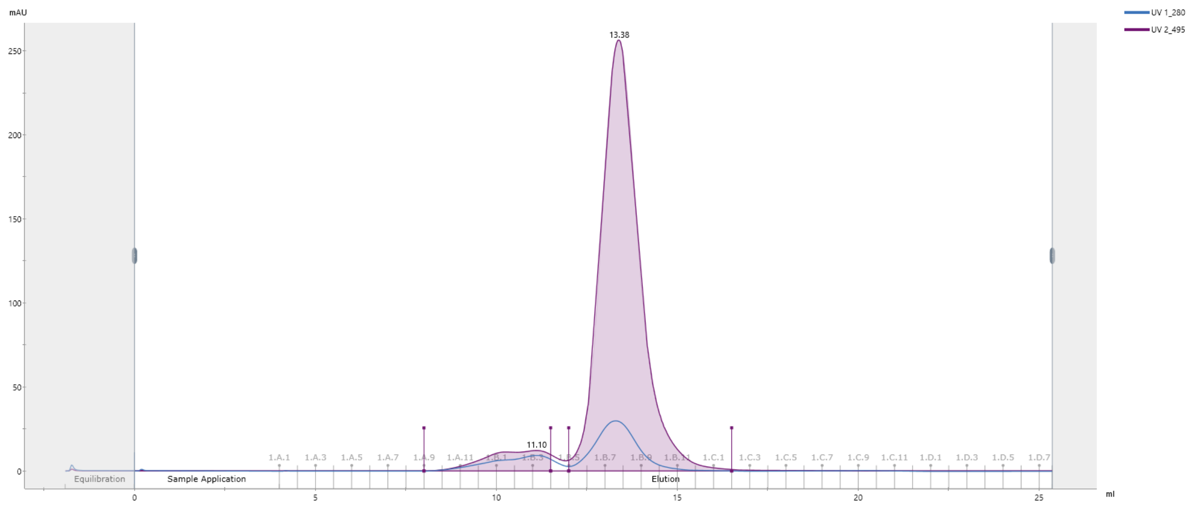


**Figure S3**. Size exclusion chromatograms of a) CD4-VioBright-Fam; b) CD8-VioBright-Fam; c) CD4-VioBright-Vio515; d) CD8-VioBright-Vio515. First peaks (8 mL – 11.5 mL) of chromatograms correspond to the desired product. Second major peaks (~ 13.5 mL) correspond to free VB-dye intermediate. Blue curve and purple curve show absorbance at 280 nm and 495 nm, respectively.

## 12. Confocal laser scanning microscopy (CLSM)

Stained cells and beads were imaged on a FluoView FV1000 from *Olympus* (Tokyo, Japan) confocal microscope. For excitation, a multiline argon ion laser (488 nm), was used. Excitation light was reflected by a dichroic mirror DM405/488 and focused onto the sample through an *Olympus* objective UPLSAPO 60xW (numerical aperture 1.2 N.A.). Emission light was recorded from 500 nm – 600 nm. The resulting images were processed using ImageJ software.

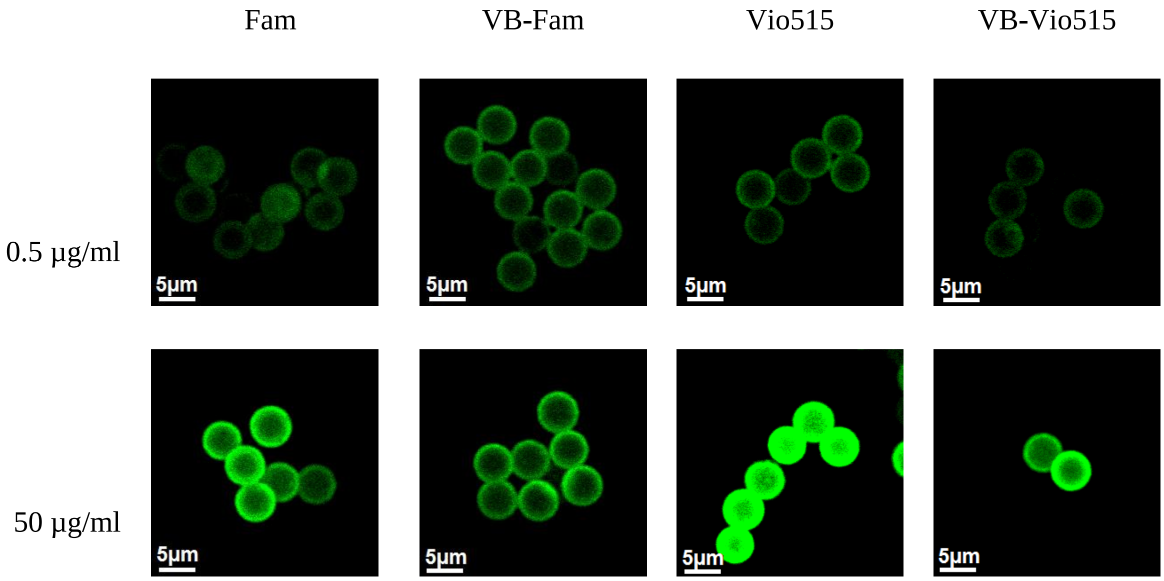

**Figure S4.** Confocal laser scanning microscopy images of the polymer beads stained with CD4 conjugates in two different titers. Top row: 0.5 µg/ml, bottom row: 5.0 µg/ml. Columns from left to right: Fam, VB-Fam, Vio515, VB-Vio515.

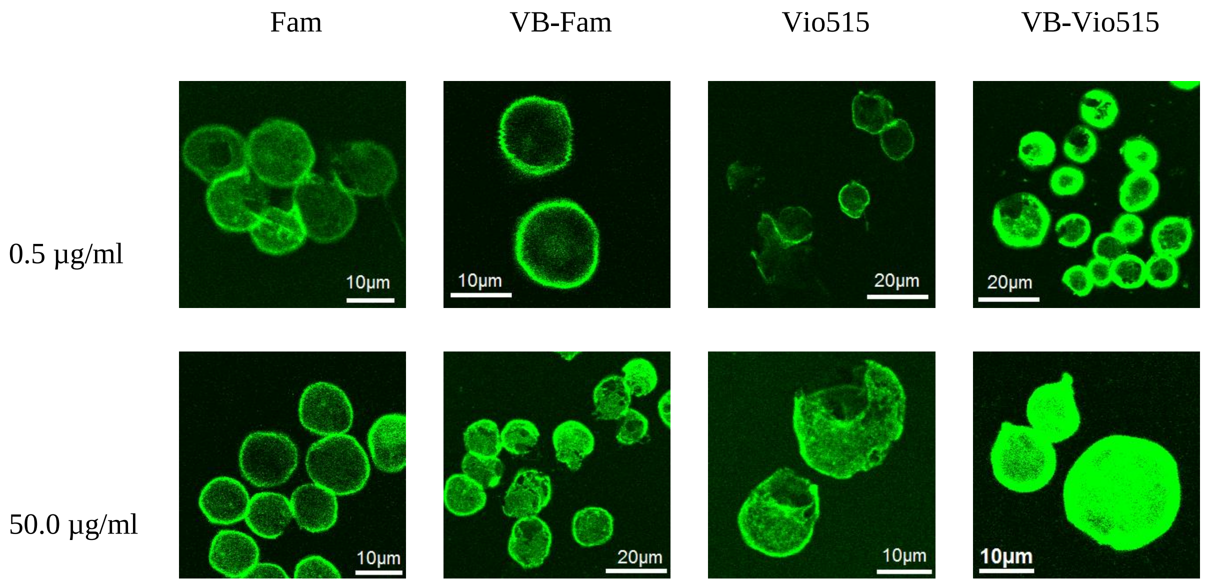


**Figure S5**. Confocal laser scanning microscopy images of SUP-T1 cells stained with CD4 conjugates in three different titers. Top row: 0.5 µg/ml, bottom row: 5.0 µg/ml. Columns from left to right: Fam, VB-Fam, Vio515, VB-Vio515.

## 13. Photostability

Zeiss LSM 710 confocal microscope from *Zeiss* (Oberkochen, Germany; excitation with an Ar-laser at 488 nm, emission detected with 470/40 nm filter settings) was used for photostability studies. The laser power was set to 5% and the gain to 675, respectively. Single cells were imaged by performing 10 scans in triplicates and the fluorescence intensities were quantified by integration of the signals derived from the fluorophore labeled membranes. The images were taken with a "Plan-Apochromat" 40x/0,95 Korr M27 objective and processed using Fiji software.

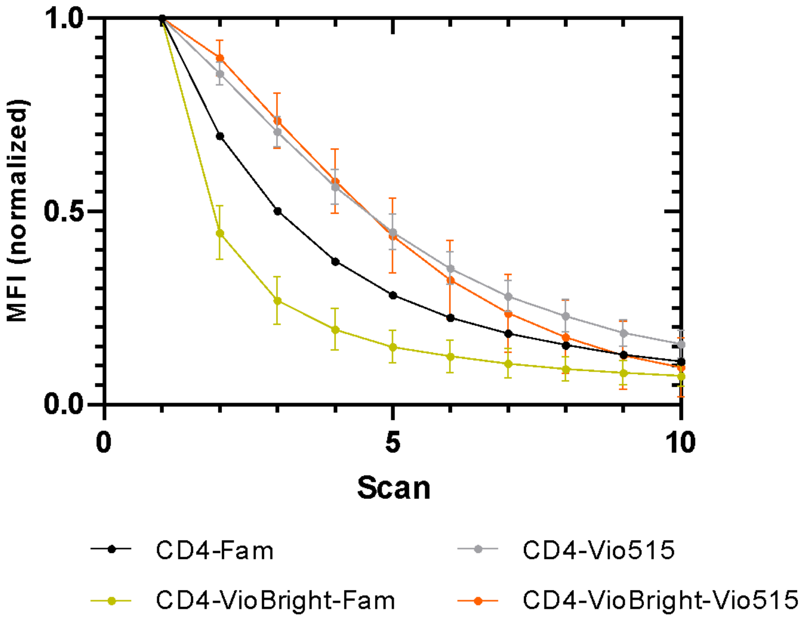


**Figure S6**. Photostability studies of CD4-dye conjugates on SUP-T1 cells.

## 14. Decay behavior of free labels in PBS buffer solution

The fluorescence decay curves of the free labels in solution were recorded as described in the manuscript.

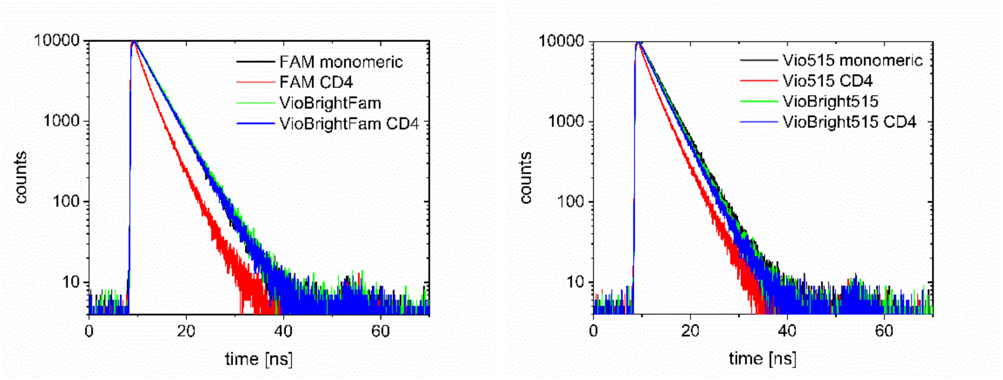


**Figure S7.** Fluorescence decay curves from lifetime measurements of free labels in PBS buffer solution.

**Table S8.** Fluorescence lifetimes determined from reconvolution fits of the recorded fluorescence decay curves (Figure S7). Monomeric species conjugated to CD4 antibodies show a reduced lifetime of the second decay component, underlining the quenching of the fluorescence of the monomeric species while the fluorescence decay kinetics are basically preserved for the multimeric labels by dye shielding provided by the multimerization process.

| | Mono-exponential | Bi-exponential | | | | |
|---|---|---|---|---|---|---|
| **sample** | $\boldsymbol{\tau}$ | $\boldsymbol{\tau_1}$**(ns)** | $\boldsymbol{B_1}$ | $\boldsymbol{\tau_2}$**(ns)** | $\boldsymbol{B_2}$ | $\boldsymbol{\tau_{int}}$**(ns)** |
| Fam monomeric | 3.89 | | | | | |
| Fam CD4 | | 1.15 | 0.0675 | 3.26 | 0.0674 | 2.71 |
| VioBright Fam | 3.98 | | | | | |
| VioBrightFam CD4 | 3.87 | | | | | |
| Vio515 monomeric | 3.75 | | | | | |
| Vio515 CD4 | | 1.02 | 0.0549 | 3.27 | 0.0785 | 2.87 |
| VioBright515 | 3.60 | | | | | |
| VioBright515 CD4 | 3.47 | | | | | |

## 15. References


[1] C. Würth, T. Behnke, J. Gienger, U. Resch-Genger, *Scientific Reports* **2023**, *13*, 6254.
